# Deciphering the impact of endoparasitic infection on immune response and gut microbial composition of *Channa punctata*

**DOI:** 10.3389/fcimb.2024.1296769

**Published:** 2024-02-27

**Authors:** Vikash Kumar, Suvra Roy, Satya Narayan Parida, Kampan Bisai, Souvik Dhar, Asim Kumar Jana, Basanta Kumar Das

**Affiliations:** Aquatic Environmental Biotechnology and Nanotechnology (AEBN) Division, Indian Council of Agricultural Research (ICAR)-Central Inland Fisheries Research Institute (CIFRI), Barrackpore, India

**Keywords:** endoparasitic infection, fish, gut microbiota, bacterial phenotype, immune response

## Abstract

Intestinal parasitic infections caused by helminths are globally distributed and are a major cause of morbidity worldwide. Parasites may modulate the virulence, gut microbiota diversity and host responses during infection. Despite numerous works, little is known about the complex interaction between parasites and the gut microbiota. In the present study, the complex interplay between parasites and the gut microbiota was investigated. A total of 12 bacterial strains across four major families, including Enterobacteriaceae, Morganellaceae, Flavobacteriaceae, and Pseudomonadaceae, were isolated from *Channa punctata*, infected with the nematode species *Aporcella* sp., *Axonchium* sp., *Tylencholaimus mirabilis*, and *Dioctophyme renale*. The findings revealed that nematode infection shaped the fish gut bacterial microbiota and significantly affected their virulence levels. Nematode-infected fish bacterial isolates are more likely to be pathogenic, with elevated hemolytic activity and biofilm formation, causing high fish mortality. In contrast, isolates recovered further from non-parasitised *C. punctata* were observed to be non-pathogenic and had negligible hemolytic activity and biofilm formation. Antibiogram analysis of the bacterial isolates revealed a disproportionately high percentage of bacteria that were either marginally or multidrug resistant, suggesting that parasitic infection-induced stress modulates the gut microenvironment and enables colonization by antibiotic-resistant strains. This isolation-based study provides an avenue to unravel the influence of parasitic infection on gut bacterial characteristics, which is valuable for understanding the infection mechanism and designing further studies aimed at optimizing treatment strategies. In addition, the cultured isolates can supplement future gut microbiome studies by providing wet lab specimens to compare (meta)genomic information discovered within the gut microenvironment of fish.

## Introduction

Intestinal parasites are among the most important infectious agents that contribute to global morbidity and mortality (Florindo et al., 2017). Infections are caused by several protozoan and helminth species, of varying severity and importance. Metazoan endoparasite infections (nematode, cestode and digenean) are very in commercial fish species, *viz*., *Thunnus albacares* (Yuasa et al., 2007) *Alectis indica*, *Carangoides chrysophrys*, *Johnius borneensis*, *Mene maculata*, *Trichiurus lepturus*, *Upeneus asymmetricus*, *U. moluccensis* (Koepper et al., 2022); *Schizothorax* species (Farooq et al., 2016); *Oreochromis niloticus* and *Clarias gariepinus* (Edeh and Solomon, 2016); *Clarias gariepenus* (Osimen and Anagha, 2021) and *Monopterus albus* (Bakti et al., 2020). However, these infections can be asymptomatic and cause mild infection and/or weight loss in fish (Koepper et al., 2022). The host-parasite relationship is extremely complex and only partially understood at this time. The prevailing consensus is that intestinal parasites have co-evolved and adapted to their hosts, a trait also suggested for prokaryotic bacteria in the gastrointestinal system (Yoo et al., 2020; Bozzi et al., 2021). Over the past few years, attention has been given to understanding how intestinal parasites interact with the host gut microbiota, or the commensal microbes (primarily bacteria) within the gastrointestinal tract, in an effort to better delineate the relationship between intestinal parasites and their vertebrate hosts. It is likely that a parasite-microbiota interaction occurs, and that this interaction may affect infection symptomatology, virulence, and outcome. This is because the gut microbiota has been shown to be pervasive and essential in fish health, including maturation and regulation of the host immune system and protection against pathogens (Fredensborg et al., 2020). The majority of intestinal parasites secrete immunomodulatory chemicals that may modify the gut microenvironment and alter the microbiota (Zheng et al., 2020). Recently, the gut microbiota was found to provide a protective barrier to suppress parasitic infections through either direct competition or indirect stimulation of host immune pathways (Giari et al., 2022). Thus, assessing the interactions between enteric parasites and the gut microbiota would provide a foundation for understanding host health, immunity, and disease susceptibility.

In Southeast Asian countries, spotted snakehead (*Channa punctata*) is a well-known indigenous farmed fish species with several health benefits. With high demand, the pressure for intensification and further expansion of culture systems has created many problems, including the limited availability of natural resources, increased environmental pollution, and losses due to disease outbreaks (Rakibuzzaman et al., 2011; Kabita et al., 2020). Parasitic infections, mainly endoparasites, are major issues in *C. punctata*, causing growth retardation and low productivity in fish farming. During endoparasitic infection, the fish are stressed and have impaired acute immune responses, which allow aquatic bacterial pathogens to concurrently attack the host and worsen the severity of the disease (Kumari Gautam et al., 2018; Tanjung and Karimah, 2019). Therefore, it is crucial to develop promising strategies to manage intestinal parasite infections. One possible way to achieve this is to enhance our understanding of the interactions between intestinal parasites and their hosts. However, screening and identification of endoparasitic infections is most often very difficult due to the associated complexity and animal sacrifice, and thus rarely done as compared to studies involving larger ectoparasitic fauna in fish.

In the present study, we explored the association between intestinal parasitic infections and the gut microbiota in vertebrate hosts. Metazoan parasites such as nematodes, which are mostly present in the tissue and abdominal cavity of fish, may affect the gut microflora, host metabolism, and immune system. However, the potential influence of enteric metazoan parasites on the gut microbiota and the consequences for the hosts are not well known in fish and remain largely misunderstood. These three-way interactions are difficult to predict, given that the parasite and gut microbiota, as well as the host species, are expected to influence the findings. To fill this gap, we conducted an experiment on fish using spotted snakehead (*C. punctata*) as a model animal to assess the effect of endoparasitic nematode infection on gut microbiota and fish health. 18S and 16S rRNA gene Sanger sequencing were used to assess nematode infection, bacterial diversity, and community composition. Further experiments were designed to investigate the phenotypic and virulent characteristics of the isolated gut microbes. We also determined the impact of nematode infection and gut microbiota interactions on host fitness by expression analysis of pro-inflammatory cytokines (interleukin 1-β and tumor necrosis factor α), immunoglobulins (Ig), and nuclear factor kappa B (NF-kb).

## Materials and methods

### Clinical observations and sample collections

From late August 2022 to October 2022, we investigated a case of severe infection in *Channa punctata* (spotted snakehead) (Length = 14.4 ± 2.2 cm, Weight = 13.38 ± 2.5 g) in the aquaculture farms of Alta Beria, Bishnupur-1, West Bengal, India. Fish farmers have reported slow growth of *C. punctata* and possible microbial infection to our institute (ICAR-Central Inland Fisheries Research Institute, Kolkata, India), which serve as a national fish disease surveillance nodal agency. During the culture site visit, we observed signs of disease, including extreme lethargy, hemorrhage, ulcer, discoloration, and redness in the fins and all over the body surface. Additionally, during the post-mortem analysis, endoparasites were observed in the abdominal cavity near the swim bladder of fish. Suspecting a microbial infection, 18 *Channa punctata* specimen including 9 infected, showing clinical signs, and 9 non-infected samples were transported to the laboratory, necropsied, and subjected to further microbiological analysis.

### Isolation and identification of parasite isolate from fish samples

For the handling and care of experimental animals, the Organisation for Economic Cooperation and Development’s (OECD) recommendations were adhered. The Central Inland Fisheries Research Institute’s Institutional Animal Ethics Committee in Kolkata, India (IAEC/2021/04) gave its approval to the procedure for using animals in the experimental setup.

In the laboratory, the samples were examined under a stereomicroscope for the preliminary identification of parasites. We calculated the prevalence of a parasite species as the percentage of hosts infected by that species and the intensity of the infection as the mean number of parasite eggs, oocysts or larvae per infected host (Bush et al., 1997). For scanning electron microscopy, specimens were prepared according to the standard method, with slight modifications (Li et al., 2016). Morphological features were used to identify different morphotypes of larval nematodes, as reported in previous studies (Shamsi et al., 2013), including the position of the excretory pore, the absence and presence of an intestinal cecum and ventricular appendix and their relative lengths, and the morphology of the ventriculus and tail tip. Later, the parasite was individually isolated and transferred to a 2 ml microcentrifuge tube for DNA isolation. DNA from the parasite was prepared by lysing the cells in 1% sodium dodecyl sulfate, 0.5 M EDTA and 10 mM Tris (pH 9.5) for 20 min at 65°C followed by incubation in 0.5 mg/ml pronase in a mixture of 0.6% sodium dodecyl sulfate, 0.5 M EDTA and 10 mM Tris (pH 9.5) for 18 h at 56°C. DNA was isolated by phenol-chloroform extraction method, precipitated with ethanol, and resuspended in Tris-1 mM EDTA 10 mM (pH 8.0) (Clark et al., 2020). PCR amplification of parasitic DNA fragments was performed using universal primers (**Supplementary Table 1**) that target ‘‘highly conserved 18S rRNA gene V4 regions’’ (del Campo et al., 2019). The extracted DNA quality was checked on agarose gel (1%) and quantified using Nano-drop (Eppendorf, Germany). The 18S rRNA gene was amplified using Gene Amp PCR system 9700 thermal cycler (Applied Biosystems, Foster City, CA). A total of 50 μl of the PCR reaction mixture consist of 5 μl of 10× PCR buffer, 1 μl of 10 mM dNTP, 1 μl of 50 mM MgCl_2_, 1 μl of 10 pmol of each primer, 1 U Taq DNA polymerase and 100 ng of isolated genomic DNA. The PCR condition includes 5 min at 94°C initial denaturation, 35 cycles of 94°C for 30 s (denaturation), 54°C for 30 s (annealing) and 72°C for 60 s (extension) and 5 min at 72°C of final extension. Amplified products were visualized on 1.8% agarose gel (Kumar et al., 2023a).

The amplified gene was sequenced in forward and reverse directions using an ABI 373xl capillary sequencer (Applied Biosystems, Foster City, CA, USA). The contig was prepared by aligning the forward and reverse sequences using a DNA baser 7.0.0. The sequence was submitted to GenBank, and a phylogenetic tree was constructed using the neighbour-joining method in MEGA X.

### Enumeration of bacteria from gut samples of *C. punctata*

The methodology developed by Guan et al. (2020) was slightly modified to determine the total cultivable bacteria abundance in the gut samples of parasitised and non-parasitised *C. punctata* (Guan et al., 2020). Briefly, the parasitised and non-parasitised group fish were disinfected with 70% alcohol, dissected under aseptic conditions, and their intestines were taken out, pooled and cut into small pieces. Tissue samples were homogenized aseptically in 10 ml distilled water for 15-30 sec at room temperature. Simultaneously, the homogenate was serially diluted with sterilized physiological saline solution, and aliquots (0.1 mL) of each dilution were spread onto Petri dishes containing tryptone soya agar (TSA) medium and incubated overnight at 28°C. Plates consisting of 30-300 CFU/ml at specific dilutions were used to count the number of colonies and calculate the abundance of bacteria in the fish gut sample.

### Identification and phylogenetic comparison of bacterial isolates

Bacterial isolates were identified using 16S rRNA PCR amplicon sequencing. The amplified gene products were sequenced in forward and reverse direction using the ABI 373xl capillary sequencer (Applied Biosystem, Foster City, CA). Assembly of the 16S rRNA sequences was performed by aligning forward and reverse sequences using DNA baser 7.0.0. The assembled bacterial 16S rRNA sequences were aligned and compared with available sequences in the NCBI GenBank using BLAST (http://blast.ncbi.nlm.nih.gov). Afterward, the sequences were submitted to GenBank for the preparation of a phylogenetic tree.

Evolutionary analyses were conducted in MEGA11 (Tamura et al., 2021) using the Neighbor-Joining method, to create the evolutionary history of recovered bacterial strains with the optimal tree (Saitou and Nei, 1987). The tree is drawn to scale, with branch lengths in the same units as the evolutionary distances used to infer the phylogenetic tree. The evolutionary distances were computed using the Maximum Composite Likelihood method (Tamura et al., 2004) and the units represent the number of base substitutions per site, with the analysis performed on 32 nucleotide sequences. Codon positions included were 1^st^ + 2^nd^ + 3^rd^ + non-coding, with ambiguous positions removed for each sequence pair using the pairwise deletion option, resulting in a total of 1444 positions in the final dataset.

### Biochemical characterization

At first, the bacterial strains isolated from parasitised and non-parasitised fish were distinguished primarily as either gram-positive or gram-negative using the Gram-staining method. The bacterial isolate was then subjected to biochemical characterization using standard techniques, including lysine utilization, urease, methyl red, esculin hydrolysis, melibiose, ONPG (β-galactosidase), phenylalanine utilization, ornithine utilization, indole, nitrate reduction, H2S production, citrate utilization, raffinose, Voges Proskauer’s (VP), glucose, malonate utilization, lactose, arabinose, xylose, adonitol, rhamnose, cellobiose, saccharose, trehalose and oxidase (KB003, Hi-media).

### Antibiogram assay

Antibiotic resistance tests were performed according to the method described by Zidour et al. (2017). The recovered bacterial isolate was grown in sterile Muller-Hinton broth at 28°C for 24h. The bacterial suspensions were diluted to 10^6^ cells/ml using sterile Muller-Hinton broth through spectrophotometer reading (Genesys 20, Thermo Spectronic). Subsequently, 100 μL of the bacterial suspension was spread onto Muller-Hinton agar plates using a sterile spreader. The plates were allowed to dry for a few seconds and five different antibiotic discs were placed on each agar plate under sterile conditions. A total of 26 antibiotic discs (Himedia, India) were used for the assay, as shown in **Supplementary Table 2**. The plates were secured with Parafilm tape and incubated for 24h at 28°C. The zone of inhibition around the disc was measured in millimeters. Bacterial sensitivity to the tested antibiotics was classified as sensitive, intermediate, or resistant according to the guidelines of the Clinical and Laboratory Standards Institute (NCCLS, 2002; CLSI, 2015). The multiple antibiotic resistance (MAR) index of each isolate was estimated according to Krumperman (1983) and the antibiotic resistance assay was performed in quintuplicate and is representative of two independent experiments.

### Virulence of isolated bacterial strains

#### Hemolysin assay

The recovered bacterial isolates’ hemolytic activity was determined using a standard procedure, with minor modifications (Zheng et al., 2021). Briefly, 5% defibrinated sheep blood was added to tryptone soy agar (TSA) media to prepare the blood agar plates. Subsequently, bacterial strains pure stock cultures were cultivated overnight in TSB at 28°C with continuous agitation. The overnight culture was diluted to 0.5 (OD600) and 2 µL of diluted suspension was spotted in the centre of blood agar plates. After 48 hours of incubation at 28°C, the plates’ clearing zone diameters were measured. Five duplicates were maintained for each bacterium and test were performed using freshly made media.

#### Biofilm formation assay

The Biofilm formation was assessed following the standard protocol developed by Tran et al., with minor modification (Tran et al., 2020; Kumar et al., 2023c). Briefly, 200 µL aliquots of bacterial suspension from the two groups previously discussed were transferred in triplicate into the wells of a sterile 96-well plate after being adjusted to an OD550 of 0.1. The only MB receiving wells served as the negative controls. To allow the biofilm to develop, the plate was incubated for 24 hours under static conditions at 28°C. To get rid of all the non-adherent bacteria, each well was then delicately washed three times with 300 µL of physiological solution (0.9% NaCl). 200 µL of 99% methanol was added to the wells, and they were then left to incubate for two hours. Later, each well’s methanol was removed and the plate was air-dried overnight. The wells were then stained for 20 minutes with 150 µL of 0.1% crystal violet, washed under a moderate stream of tap water and left to air dry. To separate the bound dye from the adhering bacterial cells, 150 µL of 95% ethanol was added to each well. After that, absorbance was determined at 570 nm using an infinite 200 Tecan plate reader (Tecan, Switzerland). The assay was performed in quintuplicate and is representative of two independent experiments.

#### Survival assay

Healthy *Labeo rohita* fingerling (length 115.52 ± 2.16 mm and weight 20.26 ± 1.02 gm) were purchased (300 nos.) from the local fish hatcheries. The fish that appeared normal and healthy, with no external clinical symptoms such as ulcer, hemorrhage, scale loss, discoloration, and redness on the body surface were used in the experiment. The fish were randomly selected (20 nos.) and screened for the possible presence of infectious microbes following a standard protocol (Johansen et al., 2006).

Briefly, the fish were screened using virulent gene specific primers with PCR methods for common freshwater microbial pathogens, including *Aeromonas*, *Pseudomonas*, *Flavobacterium* and *Edwardsiella* species. Afterwards, acclimatization of fish was done for 2 weeks in 200 L Fibre-reinforced plastic (FRP) tanks, supplied with proper commercial floating feed (Crude protein: 30%, Crude lipid: 5%) with ~ 5% of body weight fed twice a day. During the culture period, the photoperiod was maintained with a regime of 12 h light and 12 h darkness, provided with aeration (DO 6.8-7.2) and the water temperature was maintained at 27.5-28.5°C in a controlled temperature room. Subsntly, the recovered bacterial strain was cultured in 20 ml sterile Tryptone soya broth (TSB) in 50 ml Erlenmeyer flask (Himedia, India) for 24 h at 28°C. Bacterial cells pellet was collected by centrifuging for 5 min at 5000 rpm and washed thrice with a sterile normal saline solution. Afterwards, the pellets were resuspended in saline solution and the number of cells (CFU/ml) was estimated through spread plate method. The McFarland standard (BioMerieux, Marcy L’Etoile, France) was used to determine the bacterial density from optical density (OD) assuming 1.2 × 109 cells/ml at OD of 1. Later, the experimental fish were intraperitoneally injected (20 numbers/concentration) with 200 µl (10^6^ colony forming unit/ml) of bacterial suspension. Non-parasitised fish were injected with 200 µl of sterile saline solution. Afterwards, the fish were kept in a FRP tank and observed every 24 h until 168 h. To confirm Koch’s postulate, the bacteria were reisolated and identified from the liver, kidney and blood of the moribund fish.

### RNA extraction and reverse transcription

In brief, 0.1 g of liver and kidney tissue sample from parasitised and non-parasitised *Channa punctata* groups was weighed and homogenized aseptically for 15-30 s in room temperature with 1 mL chilled Trizol^®^. The homogenate was incubated for 5 min at 20°C. Then, 200 μL of chloroform was added to the homogenate, mixed vigorously for 15 min at 20°C and centrifuged at 10,000 rpm for 10 min. The upper aqueous layer was transferred to a fresh tube and 500 μL of isopropanol was added to it. The mixture was then kept at -20°C for 2 h and centrifuged again at 10,000 rpm for 10 min. The pellet obtained was washed with 75% ethanol, centrifuged at 7,000 rpm for 10 min and air-dried to remove the traces of ethanol. The RNA pellets were dissolved in 50 μL of sterile DEPC treated water. Later, the RNA was treated with DNAse I (RNase free; Thermo Scientific, India) to remove genomic DNA contamination, and its concentration (in ng/µL) and quality were obtained at an absorbance of 260/280 nm using a NanoDrop spectrophotometer (Thermo Scientific, India). RNA integrity of RNA was checked on 2% agarose gel.

Subsequently, complementary DNA (cDNA) was prepared by reverse transcription method using the RevertAid™ H Minus First Strand Synthesis Kit (Thermo Fisher Scientific). In brief, 1 µL of random hexamer primer solution were mixed with 1 µg of total RNA. Then, 8 µl of reaction mixture containing 2 µl of 0.01 mol^-1^ dNTP mix, 200 units of RevertAid™ H minus M-MuLV reverse transcriptase, 5x reaction buffer 4 µl (0.25 mol^-1^ Tris-HCl pH 8.3, 0.25 mol^-1^ MgCl_2_, 0.05 mol^-1^ DTT) and 20 units of ribonuclease inhibitor was added. The reaction mixture was incubated at 25°C for 5 min, followed by 60 min at 42°C. The reaction was stopped by heating for 5 min at 70°C, then cooled to 4°C. The complementary deoxyribonucleic acid (cDNA) samples were examined by PCR and kept at -20°C until use.

### Quantitative real-time PCR analysis

The expression of immunoglobulins (Ig) (111 bp), interleukin-1 beta (IL-1β) (86 bp), tumor necrosis factor α (TNF-α) (155 bp), nuclear factor kappa B (NF-kb) 159 bp), and β-actin (153 bp) (housekeeping gene to check for the integrity of RNA) was measured by RT-qPCR with a pair of specific primers using StepOnePlus Real-time PCR systems (Applied Biosystems) (Kumar et al., 2022; Roy et al., 2022; Kumar et al., 2023b). The amplification was performed in a total volume of 20 µl, containing 10µl of 2X Maxima SYBR Green/ROX qPCR Master Mix (Thermo Fisher Scientific), 1 µl of cDNA (50 ng), 8 µl of nuclease free water and 0.5 µl of each specific primer. Master mixes were prepared for each biological replicate of the sample in triplicate and RT-qPCR for target and reference genes was performed with a four-step amplification protocol: initial denaturation (10 min at 95°C); 40 cycles of amplification and quantification (15 s at 95°C, 30 s at 60°C, and 30 s at 72°C); melting curve (55-95°C) with a heating rate of 0.10°C/s and a continuous fluorescence measurement) and cooling (4°C). A negative control reaction was included for each primer set by omitting template cDNA. The comparative CT method (2-ΔΔCt method) following Livak and Schmittgen (Livak and Schmittgen, 2001) was used to analyze the expression levels of the target genes and was verified using the Pfaffl relative standard curve method (Pfaffl, 2002). The Log transformed 2^ΔΔCT value was subjected to a t-test, and *P* values smaller than 0.05 were considered statistically significant.

### Statistical analysis

Survival data of *Labeo rohita* fingerlings were arcsine-transformed to satisfy normality and homoscedasticity requirements, as necessary. The data were then subjected to one-way analysis of variance followed by Duncan’s multiple range test using the Statistical Software Statistical Package for the Social Sciences version 24.0. *P*-values smaller at p < 0.05. Gene expression results are presented as fold change relative to the internal control gene (β-actin). Statistical analysis for significant differences in the expression levels was performed with single-tailed Student’s t-tests using log-transformed data.

## Results

### Parasite isolation and identification by 18S rRNA gene and phylogenetic analysis

**Figure 1:**
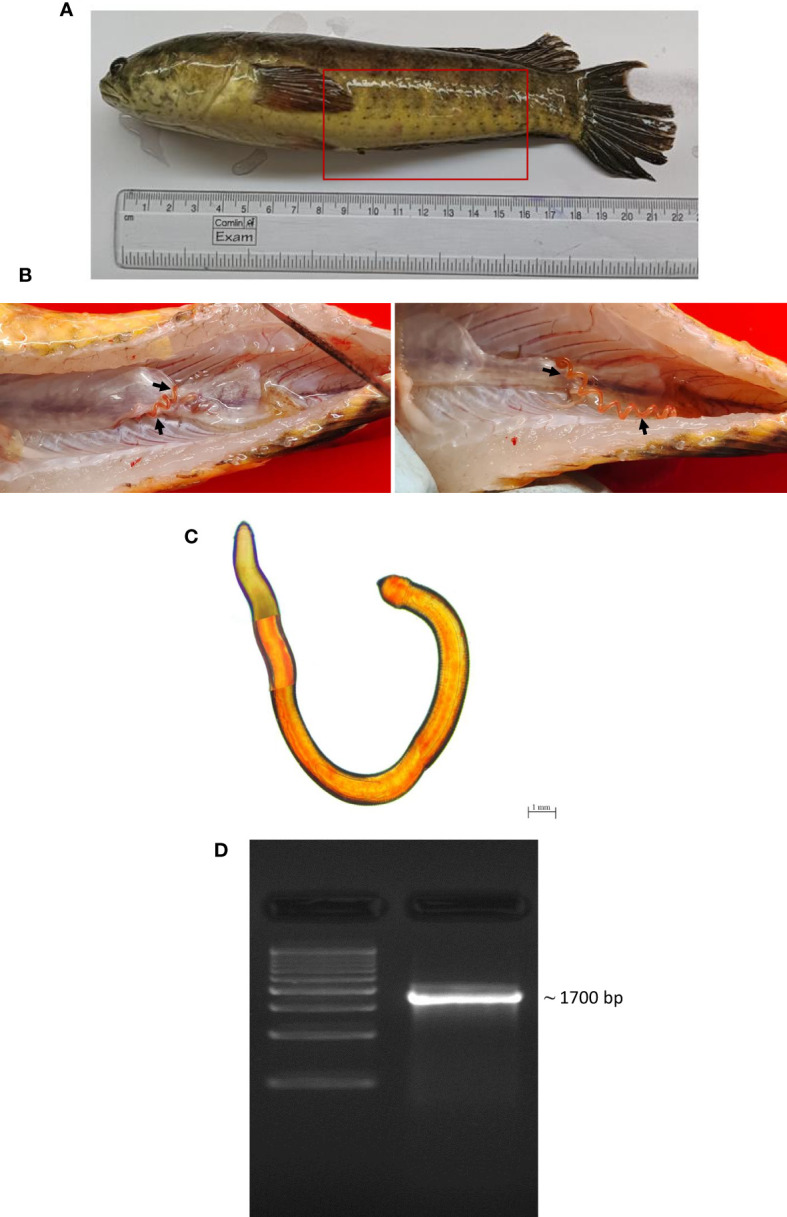
Isolation and characterization of parasite from gut of *Channa punctata.* **(A)** *C*. *punctata* sample infected with parasite and bacteria; **(B)** Presence of parasite in the abdominal cavity of fish; **(C)** Microscopic observation of parasite; and **(D)** Molecular characterization of recovered parasite sample through 18S rRNA gene analysis.

**Figure 2:**
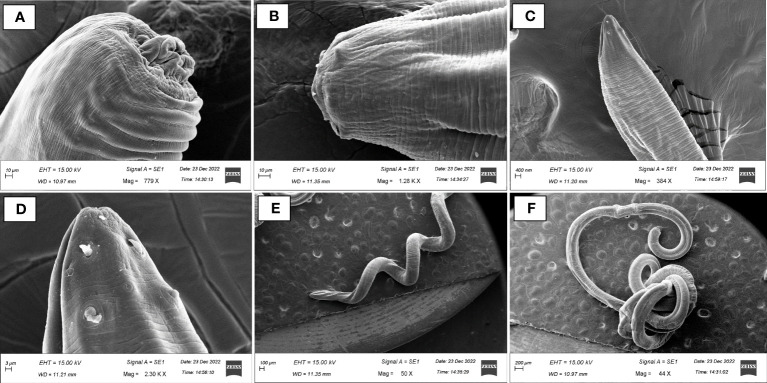
Scanning electron micrographs (SEM) of endoparasitic nematodes isolated from gut samples of *Channa punctata*. **(A)** *Aporcella* sp.; **(B)** *Axonchium* sp.; **(C, D)** *Tylencholaimus mirabilis* and **(E, F)** *Dioctophyme renale*.

**Figure 3:**
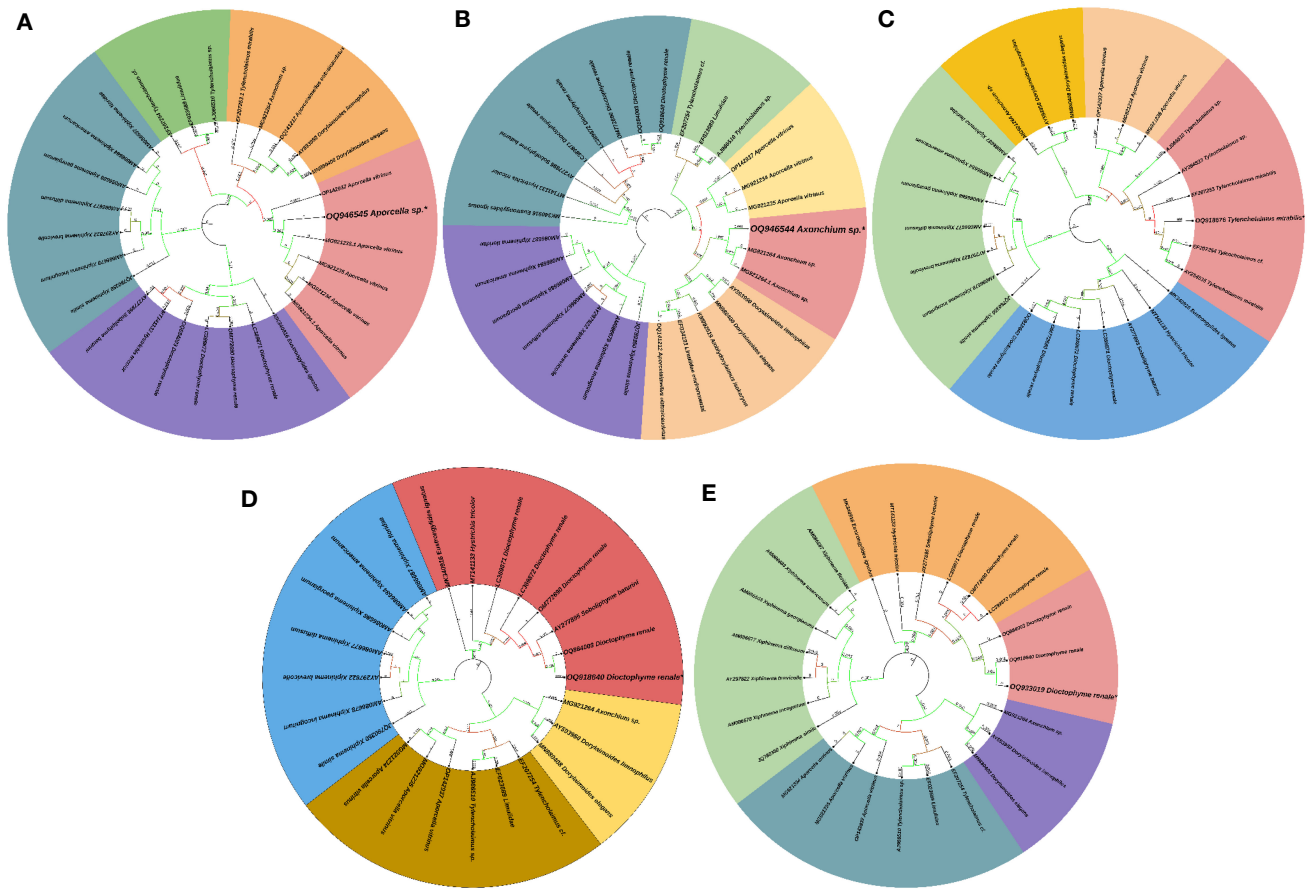
Phylogenetic tree of endoparasitic nematodes isolated from gut of *Channa punctata*. **(A)** *Aporcella* sp.; **(B)** *Axonchium* sp.; **(C)** *Tylencholaimus mirabilis*; **(D)** *Dioctophyme renale* and **(E)** *Dioctophyme renale*. Based on 18S rRNA nucleotide sequences following maximum composite likelihood method by the MEGA11 software. The numbers next to the branches indicate percentage values for 1000 bootstrap replicates. Bootstrap values above 50% are shown at the nodes. The isolates were categorized in different clusters indicated by colour shading.

Post-necropsy revealed the presence of smooth, cylindrical, relatively long worms, indicating pathogenic signs of nematode infection (**Figures 1A, B**). The % of individual tested positive for the presence of the parasite were 44.44%. The mean intensity of parasite was 1.25. The light and scanning electron microscopic analysis showed that a total of four nematode species belonging to three families were found in the gut samples of parasitised *C. punctata*. Among isolated nematodes, 24 nematode isolates were adults, and one was larvae. Adult worms with an elongated cylindrical body and anterior end with mouth opening interlocked, leaving interlabia between (**Figure 1C**). Subsequently, 18S rRNA gene sequence analysis revealed that the isolated parasitic species belonged to the families Aporcelaimidae, Belondiridae, Tylencholaimidae, and Dioctophymatidae. The nematode species included *Aporcella* sp., *Axonchium* sp., *Tylencholaimus mirabilis* and *Dioctophyme renale* (**Figures 1D**, **2**). The sequence has been submitted to GenBank and the Accession numbers are OQ946545, OQ946544, OQ918676, OQ933019, and OQ918640. The observed sequences were then subjected to BLAST-N search, and the results showed that they were highly similar to the respective group of parasitic species. A phylogenetic tree prepared from the NCBI sequence is shown in **Figure 3**.

### Effect of endoparasite on gut microbiota

The gut microbiota plays a role in host nutrient metabolism, maintenance of the structural integrity of the gut mucosal barrier, immunomodulation, and protection against pathogens (Jandhyala et al., 2015). They are also believed to play fundamental roles in the management of pathogenic microbes *via* growth suppression and antimicrobial activity (Valdes et al., 2018). Hence, we based our sampling strategy on this knowledge and hypothesized that uniqueness in morphology, color, and size of bacterial colonies are likely to be representative of a divergent bacterial group. A carefully developed culture strategy was used to target bacterial species inhabiting the gut samples of *C. punctata*. Despite the method developed, we were only able to obtain a total of 12 strains in axenic cultures from parasitised and non-parasitised *C. punctata* gut samples (**Table 1**).

**Table 1:** Bacterial isolates from gut samples of *Channa punctata*.

Experiment condition	Bacterial species	Accession number	Taxon	Gram staining	Oxygen requirement for growth
Treatment	*Providencia aicalifaciens*	OQ975752	Morganellaceae	Gram negative	Facultative anaerobic
	*Proteus valgaris*	OQ979291	Enterobacteriaceae	Gram negative	Facultative anaerobic
	*Pseudomonas aeruginosa*	OP554286	Pseudomonadaceae	Gram negative	Aerobic-facultative anaerobic
	*Proteus vulgaris*	OQ970464	Enterobacteriaceae	Gram negative	Facultative anaerobic
	*Citrobacter freundii*	OQ954767	Enterobacteriaceae	Gram negative	Facultative anaerobic
Control	*Myroides marinus*	OQ970542	Flavobacteriaceae	Gram negative	Facultative anaerobic
	*Proteus vulgaris*	OQ970545	Enterobacteriaceae	Gram negative	Facultative anaerobic
	*Providencia aicalifaciens*	OQ975745	Morganellaceae	Gram negative	Facultative anaerobic
	*Enterobacter cloacae*	OP558959	Enterobacteriaceae	Gram negative	Facultative anaerobic
	*Proteus vulgaris*	OQ970558	Enterobacteriaceae	Gram-negative	Facultative anaerobic
	*Proteus mirabilis*	OQ955591	Enterobacteriaceae	Gram negative	Facultative anaerobic
	*Proteus terrae*	OQ955602	Enterobacteriaceae	Gram negative	Facultative anaerobic

**Figure 4:**
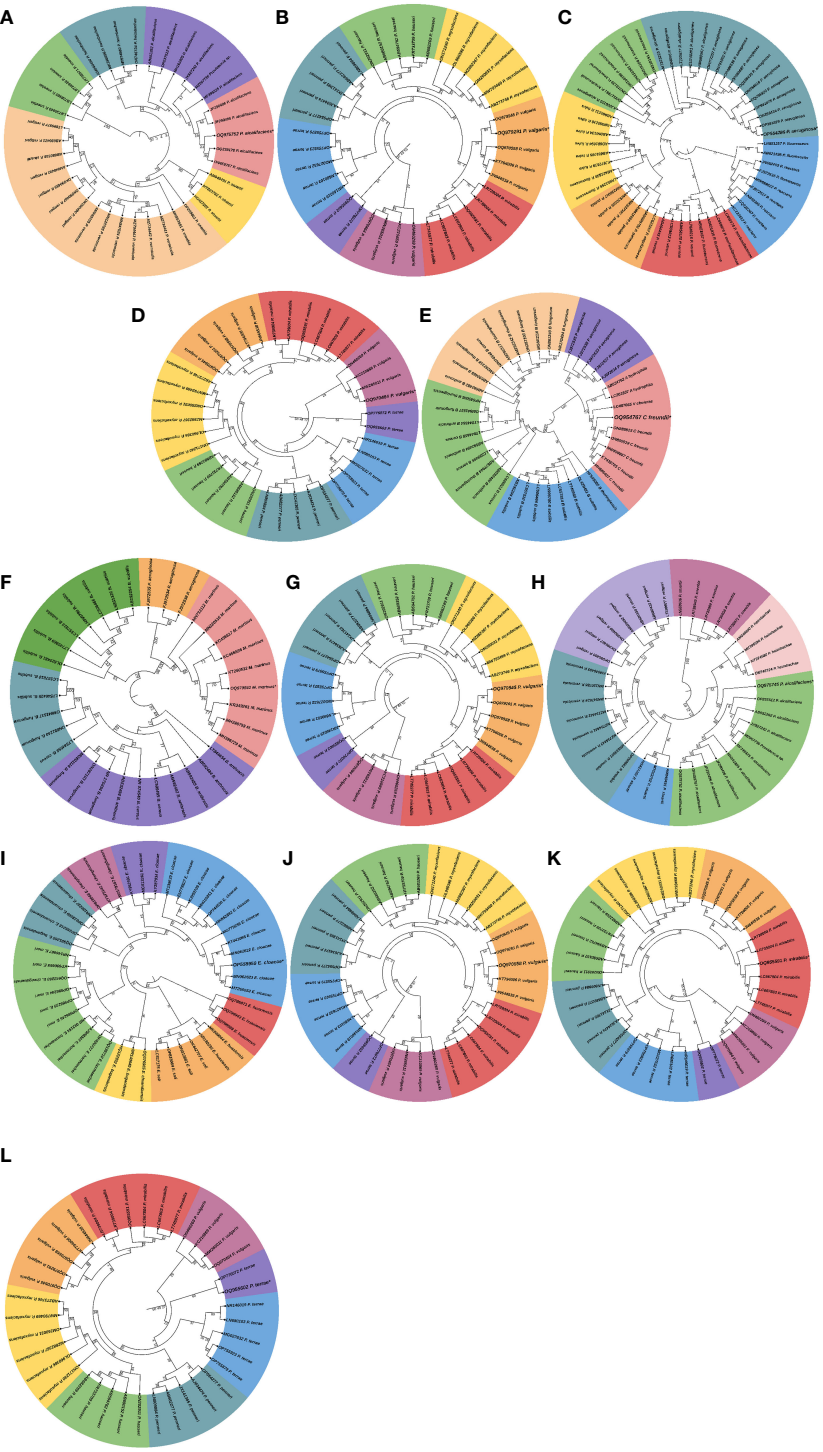
Phylogenetic tree of bacterial isolates recovered from gut samples of *Channa punctata*. **(A)** *Providencia aicalifaciens*; **(B)** *Proteus valgaris*; **(C)** *Pseudomonas aeruginosa*; **(D)** *P. vulgaris*; **(E)** *Citrobacter freundii*; **(F)** *Myroides marinus*; **(G)** *P. vulgaris*; **(H)** *P. aicalifaciens*; **(I)** *Enterobacter cloacae*; **(J)** *P. vulgaris*; **(K)** *P. mirabilis* and **(L)** *P. terrae*. Based on 16S rRNA nucleotide sequences following maximum composite likelihood method by the MEGA11 software. The numbers next to the branches indicate percentage values for 1000 bootstrap replicates. Bootstrap values above 50% are shown at the nodes. The isolates were categorized in different clusters indicated by colour shading.

Next, we assessed the phylogenetic diversity of the isolates based on 16S rRNA amplicon sequencing data. Using this strategy, we obtained preliminary information on the taxonomic clades of the recovered bacterial isolates, and the results showed that the isolates were mostly facultative anaerobes belonging to Enterobacteriaceae, Morganellaceae, Flavobacteriaceae, and Pseudomonadaceae families. The bacterial isolates in both parasitized and non-parasitized *C. punctata* gut samples included 6 Proteus species, 2 *Providencia* species and one each of *Citrobacter freundii*, *Myroides marinus*, *Enterobacter cloacae* and *Pseudomonas aeruginosa*. Most isolates clustered within Enterobacteriaceae, a clade considered normal microbiota from fish; however, some genera within this family have been associated with significant disease outbreaks. Interestingly, we also found several members of Morganellaceae (*Providencia aicalifaciens*), Flavobacteriaceae (*Myroides marinus*), and Pseudomonadaceae (*Pseudomonas aeruginosa*) in the gut samples of *C. punctata* (**Figure 4**).

### Biochemical characterization and antibiogram assay of gut bacterial isolates

The bacterial strains recovered from gut samples of *C. punctata* mostly exhibited the properties of gram-negative bacteria upon Gram staining. Additionally, the strains were observed to have unique regular and few filamentous and rhizoid morphologies on TSA plates. Biochemical tests showed that bacterial isolates from parasitised *C. punctata* samples were mostly positive for urease, methyl red, indole, xylose, saccharose, and glucose, whereas bacterial isolates from the non-parasitised group were mostly positive for nitrate reduction, indole, glucose, and oxidase. Moreover, the recovered 12 bacterial strains displayed negative results for Phenylalanine Deamination, citrate utilization, malonate utilization, Voges Proskauer’s, esculin hydrolysis, arabinose, adonitol, rhamnose, cellobiose, melibiose, raffinose, trehalose, and lactose activity (**Supplementary Table 3**).

The antibiotic susceptibility results of the bacterial strains isolated from parasitised *C. punctata* gut samples showed that the recovered isolates were resistant to multiple tested antibiotics (**Supplementary Table 4**). Approximately 80% of the bacterial isolates from parasitised gut samples had MAR index of ≥0.2, values range from 0.15 to 0.28. Moreover, the isolates from non-parasitised gut samples have the lowest range of MAR index and value ranged from 0.11 to 0.19 (**Table 2**).

**Table 2:** MAR indices and Haemolysin assay of bacterial isolates recovered from gut samples of *Channa punctatata*.

S. No.	Experiment condition	Isolates	MAR indices of bacterial strain	Haemolysin assay of bacterial strain		
			MAR value	Clear zone diameter	Colony diameter	Ratio of clear zone and colony diameter
1	Treatment	*Providencia aicalifaciens*	0.24	0	6	0
2		*Proteus valgaris*	0.19	0	6	0
3		*Pseudomonas aeruginosa*	0.15	33	5	6.60
4		*Proteus vulgaris*	0.23	28	6	4.67
5		*Citrobacter freundii*	0.28	22	7	3.14
6	Control	*Myroides marinus*	0.11	0	6	0
7		*Proteus vulgaris*	0.11	19	5	3.80
8		*Providencia aicalifaciens*	0.11	0	6	0
9		*Enterobacter cloacae*	0.11	32	7	4.57
10		*Proteus vulgaris*	0.11	0	6	0
11		*Proteus mirabilis*	0.19	0	7	0
12		*Proteus terrae*	0.15	0	6	0

### Effect of endoparasite on the virulence factor and pathogenicity of gut bacterial isolates

**Figure 5:**
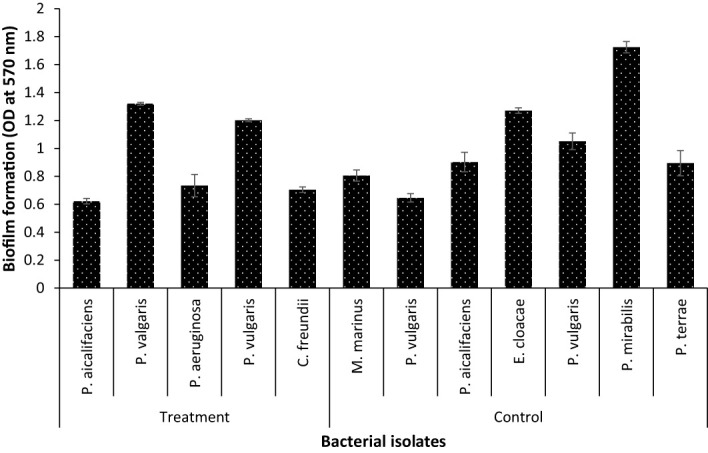
Biofilm formation assay of bacterial strain recovered from gut samples of *Channa punctata*. The assay was performed on polystyrene microtitre plates using crystal violet staining method. The error bars represent standard error of three replicates.

The hemolysin results showed that isolates from parasitised *C. punctata* samples exhibited significantly higher hemolytic activity, whereas the isolates from non-parasitised *C. punctata* had no effect on blood agar (except for two bacterial isolates showing higher hemolysin activity) (**Table 2**). Subsequently, the effects of endoparasitic nematode infection on isolated bacterial species biofilm formation were investigated. The bacterial isolates displayed significantly higher biofilm formation *in vitro* and were recovered from parasitised *C. punctata* (**Figure 5**). Lower biofilm formation values were recorded for bacteria recovered from non-parasitised *C. punctata.* In parallel, the survival study revealed that bacterial isolates from parasitised *C. punctata* samples contained *Proteus valgaris*, *Pseudomonas aeruginosa* and *Citrobacter freundii*, which induced significantly high mortality in fish fingerlings (*Labeo rohita*) (**Table 3**). In contrast, bacterial strains isolated from non-parasitised *C. punctata* gut samples were mostly non-pathogenic, and only two bacterial isolates induced significantly high mortality (*Proteus vulgaris* and *Enterobacter cloacae*) across 24-168 h time point in *L. rohita* (**Table 3**). The bacterial strains isolated from parasitised *C. punctata* gut samples had the maximum number of pathogenic bacterial isolates.

**Table 3:** Survival assay of *Labeo rohita* challenged with different bacterial isolates recovered from gut samples of *Channa punctata*.

Experiment condition	Bacterial isolates	Survival % (mean ± S.E.)						
		24h	48h	72h	96h	120h	144h	168h
Treatment	*Providencia aicalifaciens*	100 ± 0	100 ± 0	100 ± 0	100 ± 0	100 ± 0	100 ± 0	100 ± 0
	*Proteus valgaris*	100 ± 0	90 ± 3.5	90 ± 3.8	80 ± 4.8	66.7 ± 5.8	63.3 ± 2.5	56.7 ± 4.8
	*Pseudomonas aeruginosa*	6.77 ± 3.3	0 ± 0	0 ± 0	0 ± 0	0 ± 0	0 ± 0	0 ± 0
	*Proteus vulgaris*	100 ± 0	70 ± 5.7	63.3 ± 5.2	43.3 ± 3.3	30 ± 4.6	0 ± 0	0 ± 0
	*Citrobacter freundii*	100 ± 0	83.3 ± 3.9	76.7 ± 2.7	60 ± 3.8	36.7 ± 5.7	23.3 ± 2.8	10 ± 1.9
Control	*Myroides marinus*	100 ± 0	100 ± 0	100 ± 0	100 ± 0	100 ± 0	100 ± 0	100 ± 0
	*Proteus vulgaris*	100 ± 0	90 ± 3.5	90.7 ± 2.9	66.7 ± 4.8	56.3 ± 2.5	43.3 ± 1.8	36.7 ± 3.6
	*Providencia aicalifaciens*	100 ± 0	100 ± 0	100 ± 0	100 ± 0	100 ± 0	100 ± 0	100 ± 0
	*Enterobacter cloacae*	86.7 ± 3.5	70 ± 2.7	63.3 ± 1.9	56.7 ± 3.8	10 ± 1.4	0 ± 0	0 ± 0
	*Proteus vulgaris*	100 ± 0	100 ± 0	100 ± 0	100 ± 0	100 ± 0	100 ± 0	100 ± 0
	*Proteus mirabilis*	100 ± 0	100 ± 0	100 ± 0	100 ± 0	100 ± 0	100 ± 0	100 ± 0
	*Proteus terrae*	100 ± 0	100 ± 0	100 ± 0	100 ± 0	100 ± 0	100 ± 0	100 ± 0

### Interactions of endoparasite and gut microbiota on host fitness

**Figure 6:**
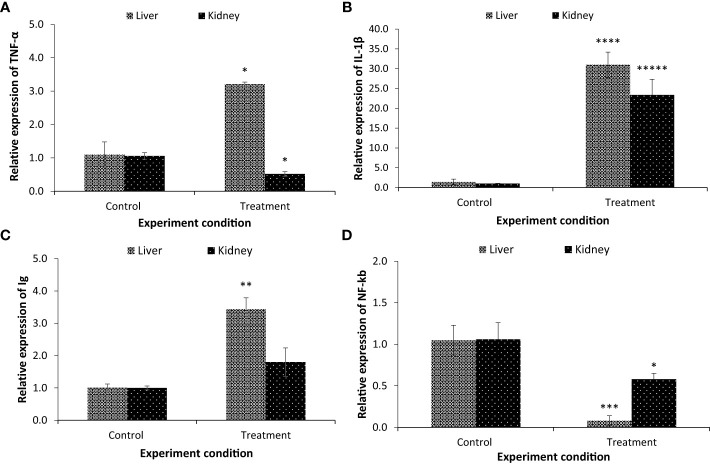
The expression of **(A)** tumour necrosis factor α (TNF-α); **(B)** interleukin-1 beta (IL-1β); **(C)** immunoglobulins (Ig) and **(D)** nuclear factor kappa B (NF-kb). Samples from both non-parasitised and parasite infected *C*. *punctata* groups were collected for gene expression assay. The expression in the non-parasitised samples of *C*. *punctata* group was set at 1. The results are the mean ± standard error (n = 3). Asterisks represents significant difference between the non-parasitised and parasitised *C*. *punctata* group. *P < 0.05, **P < 0.01, ***P < 0.001, ****P < 0.0001, *****P < 0.00001.

As there is a correlation between gut microbial composition and fish health, the *in vivo* temporal difference in the expression of pro-inflammatory cytokines (interleukin 1-β, IL-1β, and tumor necrosis factor α, TNF-α) was investigated at the transcriptional level. The results showed that the expression of TNF-α and IL-1β genes was significantly increased in the parasitised *C. punctata* group compared with the non-parasitised fish at the tested time points (**Figures 6A, B**). TNF-α gene expression was upregulated ~3 folds in liver samples, whereas downregulation was observed in the kidney. Moreover, significant upregulation of the IL-1β gene was observed in both the liver (~31 folds) and kidney (~23 folds) samples (**Figures 6A, B**).

To provide further evidence that the gut microbiota influences host immunity, the transcription of immunoglobulins (Ig) and transcription factors (nuclear factor kappa B, NF-kb) was examined. Interestingly, the expression of the Ig gene was upregulated in the parasitised group compared with the non-parasitised *C. punctata* group. The maximum values for Ig transcription were observed in the liver (3.44 folds) followed by the kidney (1.80 folds) (**Figure 6C**). Moreover, the expression of NF-kb, a protein complex that regulates multiple aspects of innate and adaptive immune functions and serves as a pivotal mediator of pro-inflammatory cytokines, was significantly downregulated in the parasitised *C. punctata* group, and the lowest values were recorded in the liver, followed by the kidney (**Figure 6D**).

## Discussion

The gastrointestinal (GI) tract of vertebrates is inhabited by a vast array of organisms including bacteria, protozoans, and helminth parasites. Increasing evidence indicates that the presence of parasitic helminths (including nematodes) alters the composition and diversity of gut microbiota in higher vertebrates (Fu et al., 2019). Moreover, there is a lack of literature on the relationship between intestinal parasites and the intestinal microbiome of fish. Few studies have presented discordant results regarding the impact of parasites on fish intestinal microbiota biodiversity (Scholz et al., 2011; Oros et al., 2015; Vasemägi et al., 2017; Kashinskaya et al., 2023). This motivated us to develop an infection model to study intestinal parasite and microbiota interactions on fish health, using *C. punctata* as a fish model. We have studied from a complex systems perspective of endoparasitic nematode infection on the host gut microbiota, focusing on the distribution of key bacterial genera. We found that the presence of nematode parasites *Aporcella* sp., *Axonchium* sp., *Tylencholaimus mirabilis* and *Dioctophyme renale* induced a significant change in microbiota network features related to bacterial virulence and host health.

The interaction of enteric parasites with the gut microbiota is an open field of study with complex interplay that reflects both the immune response and health of fish. Parasitic infection potentially causes intensive modification of the gut microenvironment through the release of harmful metabolites that could change environmental conditions, influence the growth of other organisms, and shift relative abundance levels (Calatayud et al., 2020). The “selfishness” of the winning species also prevents species from coexisting, which lowers biodiversity levels. Active growth may lead to competition for essential nutrients, counterbalancing the biotic harshness of strong competitors. Hence, assessing the relationship between parasitic species and gut microbiota could lead to the identification of biomarkers responsible for the growth and survival of fish. Here, we observed that the presence of nematode parasites had a strong influence on the microbial community structures. The parasite-infected fish included *Providencia aicalifaciens, Proteus valgaris*, *Pseudomonas aeruginosa* and *Citrobacter freundii*. The control fish included *Myroides marinus*, *P. vulgaris*, *Providencia aicalifaciens*, *Enterobacter cloacae*, *P. mirabilis* and *P. terrae*. These results are highly similar to the previously reported biochemical characteristics of *Proteus* species, *Providencia* species, *Citrobacter freundii*, *Myroides marinus*, *Enterobacter cloacae* and *Pseudomonas aeruginosa*. The present study only investigated a fraction of the huge population of bacteria, and there are likely more hitherto undiscovered microorganisms that play vital roles in the gut microbiota of *C. punctata*. Moreover, the highlights that parasitic infections simultaneously push the wheel of community succession through the course of *C. punctata*. The effect of parasitic infection on bacterial community stability is uncertain; however, as lower biodiversity often reduces stability, a higher negative network association might enhance stability (Tardy et al., 2014; Coyte et al., 2015).

The novelty of these analyses is that they allow us to find the interactions from a complex perspective, enabling us to see not only the whole system, but also how different components are affected. In this sense, when investigating the effect of parasitic infection on bacterial phenotype, we observed how the characteristic features, including the antibiogram profile, biofilm formation, and virulence characteristics of the bacterial species changed. When stressed, fish are colonized by several unique microbial species that modulate the gut microenvironment, potentially linked to the development of resistance to chemotherapy (NCCLS, 2002; CLSI, 2015). Growing resistance to antibiotics in bacteria has been documented for several decades (Davies and Dorothy, 2010), and the bacterial habitat microenvironment has recently been considered one of the main hotspots for antibiotic resistance spread. Exposure of bacteria to pollutants and/or environmental chemicals may promote the resistance genes diversity in different forms (resistant bacteria able to conjugate, free plasmids/DNA, and phage particles), enabling gene transfer probability within the bacterial communities (Balcazar, 2014; Wintersdorff et al., 2016). In the study, we found high levels of antibiotic resistance indicators within bacteria strains that thrive in nematode-infected *C. punctata*, with multi-resistance patterns to antibiotics being common among the isolates. Significantly high levels of MAR indices indicate that *C. punctata* infected with nematode parasites may harbor multidrug resistant bacteria. These results raise an important concern because endoparasitic infections are very rarely diagnosed in fish, and the presence of MDR bacteria in parasite-infected fish may spread antibiotic resistance through the food chain, with potential downstream ramifications during human consumption (Lood et al., 2017).

Various interspecies interactions that impact the growth and loss rates are reflected in the abundance profiles (Pedros-Alio, 2012; Lynch and Neufeld, 2015). Bacterial communities are “social networks,” in which individuals engage in a variety of interactions with one another, including competition for nutrition, cross-feeding cooperation, communication *via* secretion, and the detection of extracellular substances (Ratzke et al., 2020). Additionally, a phenomena known as “niche construction theory” proposes that creatures may indirectly influence other community members by altering their environment (Laland et al., 2016). To delineate how ecological factors affect interspecies interactions, we investigated how nematode parasite infection correlates with bacterial pathogenicity in fish species. Hemolysin activity and biofilm formation are target virulence factors used to determine the pathogenicity of bacterial specimens and are the criteria used to classify potentially pathogenic strains (Pandey et al., 2010). We observed that gut microbiota infected with nematodes comprises significantly higher pathogenic microbial abundance, inducing 100% mortality in model freshwater fish species (*Labeo rohita*), as well as higher hemolytic activity and biofilm formation. Moreover, non-parasitised *C. punctata* had a higher abundance of non-pathogenic isolates, exhibiting lower hemolytic activity and biofilm formation. This suggests that the ecological conditions created by parasitic infection tend to favor the proliferation of pathogenic bacteria as well as suppress the growth of possible beneficial and remedial bacteria.

Gastrointestinal parasites may modulate changes in the microbiome and induce inflammation. The gut microbiome is associated with digestion, nutrition, and health; however, alterations may result in a change in biodiversity, which can induce intestinal inflammation and increase disease susceptibility (Zheng et al., 2020; Williams et al., 2021). The fish immune response is a cascade of diverse reactions that aim to eliminate recognized foreign agents and restore homeostasis (Zhu et al., 2013). The expression of immune genes (e.g., immunoglobulins Ig, interleukin-1 beta IL-1β, tumor necrosis factor α, TNF-α, and nuclear factor kappa B NF-kb) is usually considered a sign of immune stimulation or an enhanced immune response (Roy et al., 2019; Kumar et al., 2020). Interleukin-1β (IL-1β) and tumor necrosis factor α (TNF-α), classic pro-inflammatory cytokines, play a central role in the early inflammatory response by mediating an immediate and vigorous response, inducing a number of inflammatory reactions. Immunoglobulins (Ig) and transcription factors (nuclear factor kappa B, NF-kb) are the central components of the immune system and are involved in both innate and adaptive immune defense and have many functions, including opsonization, direct killing, regulation of the immune response, and mediation of inflammation (Roy et al., 2016; Kumar et al., 2022; Kumar et al., 2023b). Moreover, in severe microbial infection the immune response is unable to cope up with pathogen mediated cellular damage resulting in overall health impairment and death of animals. In addition, in several cases, the microbial pathogens have devised ways of modulating the activity of immune response in order to gain entry in host and avoid vigorous immune response. Hence, it becomes important to understand the immune response during parasitic infection in order to understand the host and pathogen response to develop therapeutic measures and management guidelines. During parasitic infection, significant upregulation of IL-1β, TNF-α, and Ig gene were observed in *C. punctata* liver and kidney samples (except TNF-α was downregulated in kidney). While, downregulation of NF-kB was recorded in non-parasitised liver and kidneys samples of *C. punctata*. Effective early proinflammatory cytokine activity is essential for the resolution of parasitic infection-altered gut microbiota in the host. Hence, enhanced pro-inflammatory cytokine expression, which is associated with IL-1β and TNF-α responses, might be a protective modality for fish to generate antiparasitic activity and restore homeostasis (Afolayan et al., 2020). Moreover, the upregulation of Ig in the treatment group indicates that host defense plays a crucial role in host immunity and tolerance. Gene expression analysis confirmed that the enteric nematode parasite interacts with the gut microbiota and affects host immunity and health.

In this study, we have shown the importance of studying enteric parasite interactions with microbiota in terms of potential effects on bacterial phenotype, virulence, and host immunity. We have shown that isolation and characterization of enteric nematodes and gut bacteria is indeed possible when their ecological niche is sufficiently mimicked and time is allowed for slow-growing strains to propagate. By summarizing the main functional microorganisms in the gut samples of fish, we found that the family Enterobacteriaceae is the dominant family, containing microorganisms with a wide range of functions. In addition, the families Morganellaceae, Pseudomonadaceae, and Flavobacteriaceae were frequently detected in gut samples. Regarding the effects of parasitic infection on microbial diversity, we found that fish infected with nematodes had high levels of pathogenic gut microbes. While nematode infection appears to be fundamentally coupled with bacterial community diversity, parasite infection is associated with other stressors, and interactions between bacterial isolates potentially contribute, in part, to the assembly of distinct microbial communities in gut samples. This study also highlighted that host immunity plays a crucial role in the possible resolution of parasitic infections and altered gut microbiota through the expression of pro-inflammatory cytokines and immunoglobulin genes. The application of similar cultivation strategies to other under-studied bacterial species will increase the number and diversity of axenic cultures from fish samples in the future. This discovery-driven culture and identification based on biochemical and 16S rRNA amplicon sequencing techniques provide useful preliminary data that may be utilized to guide further research. Since ~ 99% of environmental bacteria are not cultivable, with countless more hitherto undiscovered microorganisms remaining in the microbial ‘dark matter’, it is unclear if the existing axenic cultures of bacterial isolates appropriately reflect bacterial diversity. Further studies are also needed to establish the gene expression profile of parasitically infected fish in order to develop biomarkers for effective management of fish health. In this regard, metagenomics, metatranscriptomics, and single-cell techniques would help gain the additional insights required to expand our understanding of the bacterial communities present within the gut samples of fish infected with enteric parasites.

## Data availability statement

The datasets presented in this study can be found in online repositories. The isolated parasites data can be found in https://www.ncbi.nlm.nih.gov/, under accession numbers OQ918640, OQ918676, OQ933019, OQ946544 and OQ946545. The isolated bacteria data can be found in https://www.ncbi.nlm.nih.gov/, under the accession numbers in **Table 1**.

## Ethics statement

All experiments were performed according to the approved animal utilization protocol was approved by Institutional Animal Ethics Committee, ICAR-CIFRI, Kolkata, India, (IAEC/2023/04) for the experimental setup. All procedures were made with maximal efforts to minimize fish suffering.

## Author contributions

VK: Conceptualization, Data curation, Formal analysis, Methodology, Writing – original draft. SR: Formal analysis, Investigation, Methodology, Writing – review & editing. SP: Data curation, Formal analysis, Investigation, Writing – review & editing. KB: Data curation, Investigation, Writing – review & editing. SD: Data curation, Formal analysis, Writing – review & editing. AJ: Data curation, Formal analysis, Writing – review & editing. BD: Conceptualization, Funding acquisition, Project administration, Resources, Supervision, Validation, Visualization, Writing – review & editing.
