# Supplementary material for: Deciphering the impact of endoparasitic infection on immune response and gut microbial composition of Channa punctata
Source: Front Cell Infect Microbiol. 2024 Feb 27;14:1296769. doi: 10.3389/fcimb.2024.1296769 (PMC10927727; doi:10.3389/fcimb.2024.1296769)
Supplement: Supplementary file 1 [file DataSheet_1.docx]

**Supplementary Table 1.** List of primers used in the study

| Target gene | Primers | Sequence (5′-3′) | Annealing temperature (°C) | Amplicon size (bp) | Reference |
| --- | --- | --- | --- | --- | --- |
| 16S rRNA | Forward  Reverse | GTTGATCATGGCTCAG  GGTTCACTTGTTACGACTT | 52 | 1414 | Kumar et al., 2022 |
| 18S rRNA | Forward  Reverse | ACCTGGTTGATCCTGCCAG  CTTCCGCAGGTTCACCTACGG | 54 | 1700 |  |
| Immunoglobulins (Ig) | Forward  Reverse | ACCTTCCCCGATGACCTGTA  GTGATCCGCCTCAAACAGGA | 49 | 111 | Used in this study |
| Interleukin-1 beta (IL-1β) | Forward  Reverse | CCGTGGAGGACAAAGACGAT  CAGCCCAGAGTCCAGTTTGT | 52 | 86 |  |
| Tumour necrosis factor α (TNF-α) | Forward  Reverse | GCCAAGGCAGCCATCCATTTAG  AAGTAGAGGCCGATTTGTGGGAT | 51 | 155 |  |
| Nuclear factor kappa B (NF-kb) | Forward  Reverse | AGGAGCAAAGATTAACATGCCAGA  GCAAAGTGAAGAGGACTGTTGC | 57 | 159 |  |
| Beta actin (β-actin) | Forward  Reverse | TGCTGTCTTCCCCTCCATCG  CAGTTGGTCACAATACCGTGC | 58 | 153 |  |

**Supplementary Table 2** Antibiotic discs used in the experiment (Himedia, India)

| **Antibiotics** | **Concentration (µg)** |
| --- | --- |
| Ampicillin | 25 |
| Tetracycline | 10 |
| Erythromycin | 10 |
| Dicloxacillin | 1 |
| Streptomycin | 254 |
| Doxycycline | 10 |
| Ofloxacin | 2 |
| Amoxycilin | 30 |
| Ceftazidime | 30 |
| Cefixime | 5 |
| Rifampicin | 5 |
| Nalidixic acid | 30 |
| Piperacillin | 10 |
| Chloramphenicol | 30 |
| Polymyxin B | 300 |
| Colistin | 10 |
| Imipenem | 10 |
| Trimethoprim | 5 |
| Ciprofloxacin | 5 |
| Netilmicin sulphate | 30 |
| Tobramycin | 10 |
| Cefepime | 30 |
| Gentamicin | 10 |
| Fosfomycin | 200 |
| Nitrofurantoin | 200 |

**Supplementary Table 3.** Biochemical characterizations of the bacterial isolates recovered from gut samples of *Channa punctatus*.

| **S.No** | **Test** | **Treatment** | | | | | **Control** | | | | | | |
| --- | --- | --- | --- | --- | --- | --- | --- | --- | --- | --- | --- | --- | --- |
|  |  | *Providencia aicalifaciens* | *Proteus valgaris* | *Pseudomonas aeruginosa* | *P. vulgaris* | *Citrobacter freundii* | *Myroides marinus* | *P. vulgaris* | *P. aicalifaciens* | *Enterobacter cloacae* | *P. vulgaris* | *P. mirabilis* | *P. terrae* |
| 1 | ONPG (β-galactosidase) | **-** | **-** | **-** | **-** | **-** | **-** | **-** | **-** | **-** | **-** | **+** | **+** |
| 2 | Lysine utilization | **+** | **-** | **-** | **-** | **-** | **-** | **-** | **-** | **-** | **-** | **-** | **+** |
| 3 | Ornithine utilization | **-** | **-** | **-** | **-** | **-** | **-** | **-** | **-** | **-** | **-** | **+** | **+** |
| 4 | Urease | **+** | **+** | **+** | **+** | **-** | **+** | **-** | **+** | **+** | **-** | **-** | **+** |
| 5 | Phenylalanine Deamination | **-** | **-** | **-** | **-** | **-** | **+** | **-** | **-** | **-** | **-** | **-** | **-** |
| 6 | Nitrate reduction | **-** | **-** | **+** | **+** | **+** | **+** | **+** | **+** | **+** | **+** | **+** | **-** |
| 7 | H_2_S production | **+** | **+** | **-** | **+** | **-** | **+** | **-** | **+** | **+** | **-** | **-** | **-** |
| 8 | Citrate utilization | **-** | **-** | **-** | **-** | **+** | **-** | **-** | **-** | **-** | **-** | **-** | **+** |
| 9 | Voges Proskauer’s | **-** | **-** | **-** | **-** | **-** | **-** | **-** | **-** | **-** | **-** | **+** | **-** |
| 10 | Methyl red | **+** | **+** | **+** | **+** | **+** | **+** | **+** | **+** | **-** | **-** | **-** | **-** |
| 11 | Indole | **+** | **+** | **+** | **+** | **+** | **+** | **+** | **+** | **+** | **+** | **-** | **-** |
| 12 | Malonate utilization | **+** | **-** | **-** | **-** | **-** | **-** | **-** | **-** | **-** | **-** | **-** | **-** |
| 13 | Esculin hydrolysis | **-** | **+** | **-** | **-** | **-** | **-** | **-** | **-** | **-** | **-** | **+** | **+** |
| 14 | Arabinose | **-** | **+** | **-** | **-** | **-** | **-** | **-** | **-** | **-** | **-** | **+** | **-** |
| 15 | Xylose | **+** | **+** | **+** | **+** | **-** | **+** | **-** | **+** | **+** | **-** | **+** | **-** |
| 16 | Adonitol | **-** | **+** | **-** | **-** | **+** | **-** | **+** | **-** | **-** | **+** | **-** | **-** |
| 17 | Rhamnose | **-** | **+** | **-** | **-** | **-** | **-** | **+** | **+** | **-** | **-** | **-** | **-** |
| 18 | Cellobiose | **-** | **+** | **-** | **-** | **-** | **-** | **-** | **-** | **-** | **-** | **-** | **-** |
| 19 | Melibiose | **-** | **-** | **-** | **-** | **-** | **-** | **-** | **-** | **-** | **-** | **-** | **-** |
| 20 | Saccharose | **+** | **+** | **+** | **+** | **-** | **+** | **-** | **+** | **+** | **-** | **-** | **-** |
| 21 | Raffinose | **-** | **+** | **-** | **-** | **-** | **-** | **-** | **-** | **-** | **-** | **+** | **-** |
| 22 | Trehalose | **-** | **+** | **-** | **-** | **-** | **-** | **+** | **-** | **-** | **-** | **-** | **-** |
| 23 | Glucose | **+** | **+** | **+** | **+** | **-** | **+** | **+** | **+** | **+** | **+** | **+** | **+** |
| 24 | Lactose | **-** | **-** | **-** | **-** | **-** | **-** | **-** | **-** | **-** | **-** | **-** | **-** |
| 25 | Oxidase | **-** | **+** | **+** | **-** | **+** | **-** | **+** | **-** | **+** | **+** | **-** | **+** |

The isolates in biochemical assay exhibited different activity are expressed as positive (+) and negative (-)

**Supplementary Table 4.** Zone of inhibition diameter (mm) by the bacterial isolates recovered from gut samples of *Channa punctatus*

| **Antibiotics** | **Treatment** | | | | | **Control** | | | | | | |
| --- | --- | --- | --- | --- | --- | --- | --- | --- | --- | --- | --- | --- |
|  | *Providencia aicalifaciens* | *Proteus valgaris* | *Pseudomonas aeruginosa* | *P. vulgaris* | *Citrobacter freundii* | *Myroides marinus* | *P. vulgaris* | *P. aicalifaciens* | *Enterobacter cloacae* | *P. vulgaris* | *P. mirabilis* | *P. terrae* |
| Gentamicin | S | S | S | S | S | S | S | S | S | I | S | I |
| Ofloxacin | S | S | S | R | S | S | S | R | S | I | S | S |
| Piperacillin | S | S | S | S | S | S | S | S | S | I | R | S |
| Amoxycillin | S | S | I | S | R | S | S | S | S | S | R | R |
| Dicloxacillin | R | R | R | R | S | R | I | R | I | I | S | S |
| Streptomycin | I | I | S | S | S | S | I | S | S | I | S | S |
| Trimethoprim | R | I | S | R | R | R | R | S | I | S | I | R |
| Colistin | R | I | R | R | S | R | R | I | I | I | S | S |
| Netilmicin sulphate | S | S | S | S | S | S | S | S | S | S | S | S |
| Fosfomycin | S | R | S | S | S | S | S | S | I | S | S | S |
| Tobramycin | S | S | S | S | S | S | S | S | S | S | R | S |
| Rifampicin | I | I | I | I | S | I | I | I | I | I | S | S |
| Nitrofurantoin | I | I | I | S | S | S | I | S | S | I | S | R |
| Cefepime | S | I | I | S | R | S | S | S | S | I | S | I |
| Cefixime | R | R | I | I | R | S | R | S | S | S | S | S |
| Kanamycin | S | R | S | S | R | S | S | S | S | I | S | S |
| Nalidixic acid | S | S | S | S | S | S | S | S | S | S | I | I |
| Tetracycline | S | S | S | S | S | S | I | I | S | I | S | S |
| Erythromycin | R | I | I | R | S | I | I | I | R | I | S | S |
| Polymyxin B | R | S | R | R | R | I | R | I | I | I | R | I |
| Chloramphenicol | S | S | S | S | S | S | S | S | S | S | R | S |
| Ciprofloxacin | S | S | S | S | S | S | S | R | S | S | S | S |
| Doxycycline | S | S | S | S | S | S | I | I | S | I | S | S |
| Imipenem | I | R | R | I | R | I | S | I | I | I | S | R |

Following the guidelines of the Clinical and Laboratory Standards Institute (NCCLS, 2002; CLSI, 2015), susceptibility of recovered strains to different antibiotics is expressed as sensitive (S), intermediate (I) and resistant (R)
